# How nurses and their work environment affect patient experiences of the quality of care: a qualitative study

**DOI:** 10.1186/1472-6963-14-249

**Published:** 2014-06-13

**Authors:** Renate AMM Kieft, Brigitte BJM de Brouwer, Anneke L Francke, Diana MJ Delnoij

**Affiliations:** 1 Dutch Nurses’ Association, PO Box 8212, 3503, RE Utrecht, The Netherlands; 2 1/ NIVEL, Netherlands Institute for Health Services Research, PO Box 1568, 3500, BN Utrecht, The Netherlands; 3 2/ EMGO+/VU medical center, Van der Boechorststraat 7, 1081, BT Amsterdam, The Netherlands; 4 Professor of Transparency in Healthcare from the Patient's Perspective, Tranzo, Tilburg University, PO Box 90153, 5000, LE Tilburg, The Netherlands

**Keywords:** Patient experiences, Quality improvement, Nurses, Nursing work environment

## Abstract

**Background:**

Healthcare organisations monitor patient experiences in order to evaluate and improve the quality of care. Because nurses spend a lot of time with patients, they have a major impact on patient experiences. To improve patient experiences of the quality of care, nurses need to know what factors within the nursing work environment are of influence. The main focus of this research was to comprehend the views of Dutch nurses on how their work and their work environment contribute to positive patient experiences.

**Methods:**

A descriptive qualitative research design was used to collect data. Four focus groups were conducted, one each with 6 or 7 registered nurses in mental health care, hospital care, home care and nursing home care. A total of 26 nurses were recruited through purposeful sampling. The interviews were audiotaped, transcribed and subjected to thematic analysis.

**Results:**

The nurses mentioned essential elements that they believe would improve patient experiences of the quality of nursing care: clinically competent nurses, collaborative working relationships, autonomous nursing practice, adequate staffing, control over nursing practice, managerial support and patient-centred culture. They also mentioned several inhibiting factors, such as cost-effectiveness policy and transparency goals for external accountability. Nurses feel pressured to increase productivity and report a high administrative workload. They stated that these factors will not improve patient experiences of the quality of nursing care.

**Conclusions:**

According to participants, a diverse range of elements affect patient experiences of the quality of nursing care. They believe that incorporating these elements into daily nursing practice would result in more positive patient experiences. However, nurses work in a healthcare context in which they have to reconcile cost-efficiency and accountability with their desire to provide nursing care that is based on patient needs and preferences, and they experience a conflict between these two approaches. Nurses must gain autonomy over their own practice in order to improve patient experiences.

## Background

In countries throughout the world, patient experiences are being monitored in order to obtain information about the delivery and quality of healthcare [1]. Patient experiences can be defined as a reflection of what actually happened during the care process and therefore provide information about the performance of healthcare workers [2]; it refers to the process of care provision [3]. In the United States [4] and many European countries [5], assessing patient experiences is part of a systematic survey programme. In the Netherlands, the government has implemented a national performance framework for comparing the quality of healthcare. This framework contains a set of quality indicators that include patient experiences. The Consumer Quality Index (CQI) is used as the measurement standard [6].

Assessing patient experiences of the quality of care not only provides information about the actual experiences, but also reveals which quality aspects patients regard as most important [7]. Many studies have been performed to analyse what patients consider essential within healthcare [8-10]. For example, a study by the Picker Institute Europe [11] revealed eight general quality aspects:

1. Involvement in decisions and respect for preferences

2. Clear, comprehensible information and support for self-care

3. Emotional support, empathy and respect

4. Fast access to reliable health advice

5. Effective treatment

6. Attention to physical and environmental needs

7. Involvement of, and support for, family and carers

8. Continuity of care and smooth transitions

The quality aspects are mostly reflected in questionnaires used to monitor patient experiences, such as the CQI [12] or the Consumer Assessment of Healthcare Providers and Systems (CAHPS) [4]. Patients are asked which aspects in receiving care are of importance and about their actual experiences [13].

Patient experiences have been identified as an indicator for evaluating and improving the quality of care [3,14]. When healthcare organisations assess patient experiences, professionals can use the results for internal quality improvements. Professionals use patient experiences and preferences to adjust their own practice and to make visible their contribution to patient outcomes [15].

Because nurses spend a lot of time with patients [16], they affect patient experiences of care [17]. Research has shown that the nursing work environment is a determining factor. It seems that when patients have positive experiences of nursing care, nurses also experience a good and healthy work environment [18-20]. A healthy work environment can be defined as a work setting in which nurses are able to both achieve the goals of the organisation and derive personal satisfaction from their work [21]. A healthy work environment fosters a climate in which nurses are challenged to use their expertise, skills and clinical knowledge. Furthermore, nurses who work in such an environment are encouraged to provide patients with excellent nursing care [21]. Research by Kramer and Schmalenberg revealed that several aspects are related to the work environment [22]. The researchers used grounded theory to identify eight ‘essentials of magnetism’ that define the nursing work environment and influence the quality of nursing care. From the perspective of nurses, the following eight ‘essentials’ are crucial in a work environment to the provision of high quality nursing care [22]:

– Clinically competent nurses

– Adequate staffing

– Good nurse–physician relationships

– Autonomous nursing practice

– Nurse manager support

– Control over nursing practice

– Support for education

– A culture that values concern for patients

### Relation between nursing work environment and patient experiences of the quality of care

The American Nurses Credentialing Center (ANCC) started the Magnet Recognition Program in the early 1990s. This programme was built upon the study carried out in 1983 by McClure et al. [23]. It is focused on improving patient care, patient safety and patient experiences by creating a good and healthy work environment for nurses. Research has shown that patient experiences in healthy work environments are significantly better [24-26].

The relationship between the nursing work environment and patient experiences was also investigated in a cross-sectional study carried out in 430 hospitals by Kutney-Lee et al. [18]. The researchers used data on patient experiences from the national CAHPS survey. The nursing work environment was measured with the PES-NWI tool, which includes items on nursing leadership and nurse–physician relationships. Data on 20,984 staff nurses were used in the study. The nursing work environment had significant relations with all ten CAHPS measures, indicating that the quality of the work environment has an influence on patient experiences of the quality of care.

This finding corresponds with the cross-sectional study by McHugh et al. [19] in which 428 hospitals and 95,499 registered nurses participated. The researchers used data from the PES-NWI and the CAHPS. They concluded that nurses’ dissatisfaction with their work environment was associated with a significantly lower quality of patient experiences.

In the RN4Cast project [20], 61,168 hospital nurses and more than 131,000 patients in Europe and the United States were questioned in a cross-sectional survey. The aim of this immense study was to determine whether the nursing work environment affected patient care. The PES-NWI was used to measure the nurses’ perceptions of their work environment. Patients’ overall satisfaction was measured with the national CAHPS survey. The perceptions of nurses and those of patients were found to be consistent, indicating that both patients and nurses had more positive experiences in hospitals with better work environments.

Although there is a relationship between the nursing work environment and patient experiences of the quality of care, it is not clear how this relationship is formed and characterised from the perspective of Dutch nurses, and which aspects in daily practice influence patient experiences. Could these aspects somehow be linked to the ‘essentials of magnetism’? Little is known about the underlying mechanisms and how these result in better patient experiences. In 2006, the Dutch government started to move towards a healthcare model of responsible consumer choice and care services competition [27]. Because of this entrepreneurial approach, healthcare organisations transformed their policy towards a cost-efficiency and productive care system (e.g. a shorter length of stay per patient) [28]. Furthermore, today’s patients tend to suffer from multiple disorders or illnesses, which results in a higher complexity of care and an increased nursing workload. The increasing complexity of patient care requires well-trained nurses who are capable of creating a safe and patient-centred environment [29]. In 2011, the Netherlands Institute for Health Services Research conducted a literature study to investigate the roles and positions of nurses in Belgium, Germany, the United Kingdom, the United States and Canada, and found differences in levels of education and nursing job profile or job description in all five countries [30].

Given the circumstances and changes with which Dutch nurses are confronted, it is important and relevant to examine and comprehend their views on how their work and work environment contribute to positive patient experiences.

## Methods

### Aim of study

The aim of this study was to understand from the perspective of nurses how the nursing work environment is related to positive patient experiences.

### Research question

The central research question was: According to nurses, which elements of their work and work environment influence patient experiences of the quality of nursing care?

The sub-questions were:

– Are these elements related to the eight essentials of magnetism?

– What is the mechanism by which these elements lead to better patient experiences?

### Research design

A phenomenological approach was applied to explore areas about which little is known or to gain an understanding of specific areas. Phenomenology is the study of subjective experience, feelings and behaviours of people [31,32].

### Sample size, composition and data collection

To gain a deeper understanding of the influence of the nursing work environment on patient experiences, we conducted four focus groups. The purpose was to elicit ideas, thoughts and perceptions from nurses [31] about patient experiences and how nurses can improve those experiences. We recruited participants by purposeful sampling, using the following criteria:

– Participants must be employed as registered nurses or certified nursing assistants.

– Participants must have worked as nurses for at least two years.

– Participants must be operative in mental health care, hospital care, home care or nursing home care.

Nurses are active in various settings and every setting has its specific dynamics. By gaining insight into their perspectives, we were able to compare possibly different views. In addition, we obtained an overall view of the total healthcare system.

The organisations we recruited are participating in a Dutch programme called Excellent Care. The programme is based on the eight essentials of magnetism and focuses on creating a dynamic, inspiring and innovative nursing work environment in order to improve the quality of care. We asked the programme director of each organisation to recruit nurses for the focus groups. A total of 26 registered nurses participated. Each focus group consisted of 6 or 7 registered nurses in mental health care, hospital care, home care and nursing home care, respectively. The nurses described their perceptions and views with respect to their own areas of expertise.

Each focus group discussion was led by two researchers. One researcher facilitated the interview, and the other had an observing role and monitored the process. After each focus group, the researchers evaluated and critically reflected on the process in order to examine the quality of the meetings. This investigator triangulation allowed the dissection of possibly different views.

The researchers used an interview guide with predefined topic areas (Table 1, topic list). The sequencing of questions depended on the process of the group and the responses of the informants.

**Table 1:** Topic list

**Questions:**	**Topics:**
Which elements in daily nursing practice influence patient experiences?	Clinically competent nurses
In what way do nurses effect experiences of patients?	Adequate staffing
What are inhibiting or facilitating factors?	Nurse-physician relationship
	Autonomous nursing practice
	Nurse manager support
	Control over nursing practice
	Support for education
	A culture that values concern for patients

Each focus group lasted two hours. The researchers explained the procedures and introduced the topic to be debated. When the informants were discussing certain topics, the researchers applied a non-directive approach because of the dynamics of the group and the different perspectives that were being examined. When certain views were polarised, the researcher stimulated the discussion by introducing a new question or topic. All conversations were digitally recorded and then transcribed to improve transferability.

### Ethical considerations

This was a qualitative study in competent subjects without any intervention. It did not involve any form of invasion of the participant's integrity, and in such cases no approval by an ethics committee is required in the Netherlands (according to the Medical Research Involving Human Subjects Act; see ccmo-online.nl). All respondents received written and verbal information about the aim and content of the study. Study participation was voluntary. Data were analysed in an anonymous way and the results were non-traceable to individual participants.

### Data analysis

The transcribed data were open coded and categorised. Several themes were extracted by organising and structuring the categories. During the analytical process, interview fragments were constantly compared. The literally transcribed interviews were reviewed several times to check whether elements might have been overlooked. The final analysis was presented to the participants and they were asked to comment on the contents. This member check helped to determine whether we had adequately understood and interpreted the data. The analytical procedure and findings were discussed within the research team to improve the quality of analysis. MaxQDA software was used to support the coding ordering analyses.

## Results

The sample consisted of 26 registered nurses (6 male and 20 female nurses). The mean age of the participants and the mean length of nursing experience varied per focus group, as shown in Table 2 below.

**Table 2:** Demographics of the participants

**Focus group**	**Age (mean)**	**Gender**	**Length of nursing experience (mean)**
Hospital care	34 years	3 male, 3 female	13 years
Mental health care	36 years	2 male, 4 female	16 years
Nursing home care	51 years	8 female	19 years
Home care	46 years	6 female	22 years

Participants formulated several facilitating elements that they consider fundamental to improving patient experiences of the quality of care. They also mentioned such inhibiting factors as cost-effectiveness and transparency and accountability goals. These factors prevent them from improving patient experiences (Table 3).

**Table 3:** Facilitating and inhibiting elements

**Facilitating elements**	**Inhibiting factors**
Clinically competent nurses	Cost-effectiveness policy
Collaborative working relationships	Transparency and accountability goals
Autonomous nursing practice	
Adequate staffing	
Control over nursing practice	
Managerial support	
Patient-centred care	

Both facilitating elements and inhibiting factors are elaborated below.

## Facilitating elements

### Clinically competent nurses

Participants stated that in order to act in a professional manner, nurses need to have certain competencies, namely social skills, expertise & experience, and priority setting.

#### *Social skills*

Participants stated that social skills are an important competency to create a trustful care relationship. They indicated correct behaviour and attitude, composure, making time for patients, and listening and having empathy as essential nursing competencies. According to participants, these social skills convey a sense of commitment to the patient and play a major role in meeting patient expectations.

*Nurses must have the ability to develop and maintain good relationships with patients. For patients, nursing care is about being heard and seen. Knowing that you’re in safe hands. You allay their fear and uncertainty. You give patients confidence and hope in return. You offer them several options from which they can choose. Someone who is dependent, and does not know what will happen, is more suspicious and anxious.* (Respondent 21, hospital focus group)

#### *Expertise & experience*

Participants mentioned three key aspects related to expertise, namely knowledge, technical skills and communicative capabilities. According to participants, the first key aspect means that nurses must have substantive knowledge related to the nursing profession. They indicated that nurses should maintain and follow both existing developments and new insights. According to participants, nurses must continually invest in nursing knowledge and education. In their view, nurses ought to offer state-of-the-art interventions or activities that are in line with the agreed nursing policy.

As a second key aspect related to expertise, participants indicated that nurses must have technical skills in order to provide effective and safe care.

The third aspect mentioned by participants is that nurses must have communicative capabilities. Participants said that nurses serve as spokespersons for patients who are often in vulnerable positions. They stated that nurses are easily accessible and can act as a link between the patient and other professions. According to participants, nurses can use the right substantive arguments on behalf of a patient’s interests or needs. Participants mentioned that this expertise is important for patients because it is related to the quality of care.

*If you can answer a care-related question, it gives the patient a certain peace of mind. It signals: she knows what she's talking about. I notice that patients really appreciate it when I share knowledge and offer them information that at the time they don’t yet have. Only then can patients make decisions about their own care.* (Respondent 15, nursing home focus group)

In addition to substantive expertise, participants stated that nursing experience is also of influence. According to them, a junior nurse has too little experience to respond creatively to sometimes complex care situations. However, according to participants, junior and senior nurses can learn from each other: they should work as a team and collectively pursue their common objectives. In their view, experience is gained through practice. According to participants, this can be characterised as 'expertise'.

*When you suspect someone is contemplating suicide, you need to know how serious this is. Is it just a cry of “I'm not feeling well” or are these serious thoughts? Has the patient already made plans, does the patient have a death wish, or is it an impulsive thought? In that sense you need to reflect on the signals very carefully. You can only learn this from practice.* (Respondent 1, mental health care focus group)

#### *Priority setting*

As stated by participants, various activities can occur simultaneously during the daily care of patients. According to them, nurses should assess what care is needed and then flexibly coordinate diverse actions with each other. In the view of participants, prioritisation is about the organisation of nursing care. Patients need nurses who have clinical experience in order to coordinate care. Nurses must decide what choices to make, what is urgent and what is important. Those choices influence patient experiences.

*Prioritisation is very important. It means that you have to coordinate the daily care and decide which activities have priority. Patients sometimes have to wait for help. If you’re in a hasty mood, you transmit that feeling to patients. It shows immediately. The restlessness affects the other patients.* (Respondent 18, nursing home focus group)

Participants said that patients sometimes have to wait before they are taken care of, or that nurses are not immediately available to answer questions or deal with problems. According to participants, patients do not always obtain the right and needed care, especially when the nurses’ workload is high.

### Collaborative working relationships

According to participants, it is important to develop and maintain collaborative working relationships with professionals, including those in their own field. In the view of participants, collaborative working relationships exist when all the involved professionals interact and operate in a complementary manner, and show mutual respect that is based on knowledge and expertise. Participants stated that all professionals need to discuss and influence patient care on the basis of their own expertise. Participants believe that problems will be solved sooner when ideas and thoughts are exchanged. In their view, it is about sharing information and communication. As stated by participants, communication and aligning with each other is needed so that no conflicting information is given and uniformity in care or treatment is provided. This generates, according to the participants, composure and clarity towards patients.

Participants believe that collaboration and communication affect how patients experience the quality and effectiveness of care.

*We have a patient who is very compulsive. We made agreements about how to approach and handle this patient. We continually need to communicate with each other, physicians, psychologists, nurses. Clear communication is so important, and I miss that sometimes. When you have good relationships it is easier to review and discuss the treatment administered. It will not only increase your knowledge, but also be helpful in the communication with the patient and his family. It’s easier to explain why the specific treatment is being deployed.* (Respondent 5, mental health care focus group)

### Autonomous nursing practice

Participants in all four focus groups stated that the scope of practice for which they are accountable influences patient experiences. The scope of practice, according to them, means that nurses can control their own work related to patient care and can make independent decisions about patient outcomes based on clinical judgements. Participants therefore believe it is essential to monitor and measure outcomes, as long as the monitoring is directly related to patient care. However, participants indicated that they did not have insight into care results obtained from assessments.

*We participate in an annual national prevalence survey. We have to fill out a lot of forms. It’s an administrative burden and takes a lot of time – time we can’t spend on patient care. We get a pile of papers, screen patients and register them. It doesn’t contribute to the quality of care because we never get any feedback. And what does one measurement tell us? It doesn’t inform us whether we are doing well or not. I do not believe that.* (Respondent 12, home care focus group)

According to participants, there is no policy to improve patient experiences on the basis of the information derived from assessments. Participants could not indicate whether the interventions deployed are actually leading to desired nursing care results, including patient experiences. Participants feel they have insufficient autonomy to influence this process.

### Adequate staffing

Participants stated that the number of nurses available influences how patients experience the quality of care. Although they could not indicate what number they consider sufficient, they think that a sufficient nurse staffing level is linked to team composition or staff mix. For instance, participants indicated the proportion of registered nurses to student nurses, or the number of different nurse qualification levels in one team. Participants stated that several tasks and assignments have been transferred to nurses with a lower qualification in order to work as efficiently as possible and to achieve higher productivity. As a result, participants believe that nursing care is, in general, increasingly developing in the direction of task-centred care in which different working methods are applied. According to them, this affects patient experiences of the quality and effectiveness of nursing care.

*Nurses provide care within certain theoretical frameworks that are designed to increase the self-reliance and self-management of the patient. Nurse assistants have a more practical focus and take over patient care at a point when they should not. These two ways of working are confusing for patients. And we think 'How come the patient is made to feel so nervous?’ and afterwards we notice two contradictory ways of working.* (Respondent 3, mental health care focus group)

As stated by participants, a sufficient nurse staffing level determines whether patient wishes and needs are met. According to participants, an insufficient deployment of nursing staff has a direct negative impact on patient experience.

*I work alone in a group. For example, when I’m in the bathroom with a patient, the other patients are alone. So I have to keep my eyes and ears open and must respond to what occurs. And that is not always easy. I constantly think: I must check if everything is all right. Because I’m responsible for the other patients. I always leave the bathroom door partly open, so I can see and listen to what is going on in the living room. I provide patient care too hastily. My patients obviously feel that.* (Respondent 17, nursing home focus group)

### Control over nursing practice

The participants stated that control over nursing practice means that nurses are involved in nursing policy or nursing issues. In their view, nurses are not always in charge and cannot always make their own decisions about nursing issues. Participants feel that this affects the quality of nursing care.

*In the past, I always made my own schedule. Now we have planners and they don’t have any experience with care. Efficient planning is more important than patient-centred planning. It doesn’t matter whether it suits the patient. The patient should be scheduled later if it fits better in the planned route.* (Respondent 9, home care focus group)

The participants stated that if nurses were more involved in the development of nursing policies, this would have a positive influence on patient care. According to them, they would be able to reflect upon and discuss nursing issues related to the quality of patient care, which would improve the quality of care.

### Managerial support

Participants indicated that a manager should pay attention to the team spirit and unity. In their view, a manager must be able to handle conflicts, and also be visible and approachable. Participants said that they believe that a manager should ask the opinion of nurses; therefore, in their opinion, regular contact is important.

A manager, according to the participants, must be able to create the right conditions and have the logistical ability to ensure continuity of care. In their view, this means arranging sufficient personnel, replacement staff and succession planning.

Participants find that managers critically examine the deployment of personnel. According to them, the nursing staff mix has drifted towards a model whereby higher-educated nurses are replaced with lower-educated ones. They noted that management is tied to a system that is dominated by controlling costs. Thus in their view, nurses may want to provide a patient with a specific form of care, while management limits care to a maximum number of minutes based on budgetary considerations. According to participants, nurses regularly experience a tension with management in shaping care that meets patient expectations.

*We want to provide certain care, but that’s at the expense of something else. If we do one thing, we can’t do another. For instance, we plan 30 minutes for patient care. When a patient wants to go outside for a walk, this will cost him 10 minutes of this total time. So we really have to negotiate with the patient or his family. This leads, of course, to lots of misunderstandings. I understand that feeling.* (Respondent 13, nursing home focus group)

### Patient-centred care

According to participants, the focus of nurses is the provision of patient-centred care. They define this as nursing care that is focussed on patient needs and preferences and is intended to increase patient self-management and encourage improved health and recovery.

As participants stated, nurses are the first points of contact for patients. In the participants’ view, they are often with the patient for 24 hours/7 days a week (except for home care) and gather large amounts of information about them. They think that direct contact with patients is crucial to building and maintaining a relationship of trust. The participants believe that high quality nursing care is achieved when patients feel heard and understood, consider themselves to be in safe hands and know that their care problems have been noticed. This, according to the participants, results in positive patient experiences.

*We listen to the patient and talk to him. We immerse ourselves in his background. What is important, how he copes and handles care problems. Based on this knowledge, we present the patient with a number of options so that he can decide upon a solution for his care problems.* (Respondent 8, home care focus group)

### Inhibiting factors

The participants talked about two inhibiting factors that prevent them from improving patient experiences: cost-effectiveness and transparency & accountability goals.

#### *Cost-effectiveness*

Participants stated that organisation policy is focused on the efficient and effective deployment of people and resources. They mentioned the transfer of tasks to less well qualified nurses in order to work as efficiently as possible and to achieve higher productivity. In their view, care is more and more standardised. At the same time, they noted that care has become increasingly complex. According to them, patients are generally older and have multiple age-related comorbidities. The participants experience an increasing workload and work-associated pressure.

*In recent years, patient turnover has increased. It means that patients are discharged quicker. As soon as they recover, they’re sent home. However, patients sometimes also have chronic disorders. I sometimes think it is irresponsible [to send these patients home so quickly]. Patients get less attention because the work pressure is high.* (Respondent 22, hospital focus group)

#### *Transparency & accountability goals*

Participants reported an increasing administrative workload to account for the quality and costs of care.

*So many forms. Entering the data means a double administrative workload. We use different programs. We first have to register in program X. Then we have to register our measurements and enter all kinds of codes in another program. Log in and log out. The registrations and coding are needed for the government and health insurers. It is not always patient related and does not inform us about the health status of patients.* (Respondent 23, hospital focus group)

The administrative workload is, according to participants, out of balance. They said that this means that monitoring and registration is aimed not at improving nursing care, but at serving an external accountability goal to inform health insurers and the government.

The participants stated that they have little autonomy to change this policy. According to them, monitoring care results should help nurses to improve their own practice. For them, it means that nurses can reflect upon and discuss nursing issues related to quality of patient care, including the results of patient experiences.

## Discussion

We interviewed 26 nurses working in various Dutch healthcare settings in order to ascertain their views on how their work and their work environment contribute to positive patient experiences. Using an open approach, we obtained insights into their perceptions and noted what they said. Participants stated that a diverse range of elements are essential to providing high-quality nursing care. When these elements are incorporated into daily nursing practice, the participants expect it will result in more positive patient experiences of nursing care. The elements are: clinically competent nurses, collaborative relationships, autonomous nursing practice, adequate staffing, control over nursing practice, managerial support and patient-centred care.

One of the sub-questions was whether the identified elements are related to the eight essentials of magnetism defined by Kramer and Schmalenberg [22]. We found that they are. The essential of magnetism ‘nurse–physician relationships’ is, in our opinion, not totally applicable in a modern healthcare system. Although physicians are represented in all settings, also other professionals, such as psychologists, social workers or physical therapists, are part of a healthcare team. The participants stated that a good relationship must be based on collaboration and clear communication not only with physicians, but with all involved healthcare workers. The participants stated that patient wellbeing must be the common aim of all the involved professionals and that communication and collaboration must support this shared goal. We therefore replaced ‘nurse–physician relationships’ with ‘collaborative working relationships’.

### Competing policies in the nursing setting

The other sub-question concerned mechanisms by which these elements lead to better patient experiences. By analysing the data it became clear that nurses operate in a complex healthcare context. These different views control the manner in which nurses can practise their profession. We noticed that nurses are confronted with organisation policies that are focussed on cost-efficiency, transparency and accountability goals. According to participants, this has led to a more productive care system. It also became clear that nurses flourish within a patient-centred care system. Such a system supports individual patients in their need to make decisions and participate in their own care. This means that organisations should facilitate a culture where nurses can professionally support patients by practising high-quality nursing care [33].

Each view is defendable on its own, but collectively they contradict each other. The context in which nurses work is almost paradoxical: they have to offer patient-centred care in a standardised and productive care system.

In the Dutch context, healthcare insurers, the government and healthcare providers are responsible and accountable for providing good quality care. However, these parties have different foci. Each year, healthcare insurers make agreements with healthcare providers about which care will be delivered. These agreements are defined in a healthcare procurement contract [28]. Individuals who legally live in the Netherlands are obliged to take out individual health insurance [27]. In order to make well-considered choices, individuals need to be informed about the quality of care provided by healthcare workers. Healthcare insurers are therefore driven by accountability goals, because they need to determine whether healthcare organisations or professionals meet the minimum standard of performance, as agreed upon in the healthcare procurement contract [34]. The government is the supervisory authority that ensures the proper functioning of the healthcare system and is therefore responsible for the transparency process [35]. In the Netherlands, a national performance framework for comparing the quality of healthcare is implemented under the supervision of the government [36]. This framework contains a set of quality indicators and related measures, including patient experiences [6,37]. Healthcare insurers and the government collect data for external accountability goals [38]. Healthcare providers and professionals themselves are also responsible for the quality of care. Their aim is more internally driven, namely to improve the quality of care and to make visible their contribution to patient outcomes [39,40]. However, our research showed that nurses do not receive feedback on their scores and they are not aware that they could – and even should – use these data to monitor and improve the quality of their work.

It could be argued that the dominance of cost-effective policy and transparency determines the manner in which nurses can practise their profession and that this influences patient experiences of care. Ancarani [41] showed that patient satisfaction was negatively associated with management-controlled wards that are under pressure to produce. Open, collaborative, innovative wards and wards that are focused on the welfare and involvement of nurses and that provide supervisory support and training were positively associated with patient satisfaction. This confirms that the environment in which nurses operate influences patient experiences of the quality of care. This corresponds with the findings of our research, in which participants stated that the dominance of policies focussed on cost-effectiveness and transparency lead to more pressure to produce and a high administrative workload. The participants feel that they have insufficient autonomy to influence this policy.

### Strong nursing practice

To incorporate the identified elements into nursing practice, cost-effectiveness, transparency and patient-centred care policy need to be connected. For example, the registration and monitoring of outcomes should be used not only to quantify achievements against transparency goals, but also for overall nursing quality improvement. Nurses should be able to decide which issues are of importance to improve patient care.

Connecting the different policies requires the participation and commitment of both nurses and nursing management. Nurses need to be challenged to shape their own environment and create a strong nursing practice [42], which will result in more positive patient experiences [43].

### Limitations of this study

We conducted four focus groups, one each with nurses in mental health care, hospital care, home care and nursing home care. Although we gained a broader insight into the perspectives of nurses, every sector has its specific dynamics and context. Therefore, one focus group per sector might have been insufficient. However, we reached data saturation as new information did not appear and similar themes emerged within the focus groups.

This study was limited to nurses, but to fully understand the nuances of this relation, it might be interesting to analyse patients’ views.

## Conclusion

The knowledge obtained from this research has resulted in a better understanding of how nurses regard their role in achieving positive patient experiences. From the viewpoint of the interviewed nurses, several elements are essential in relation to patient experiences of the quality of nursing care: clinically competent nurses, collaborative working relationships, autonomous nursing practice, adequate staffing, control over nursing practice, managerial support and patient-centred culture. These elements correspond to the eight ‘essentials of magnetism’. If these elements are incorporated into the nursing practice, it will most likely result in more positive patient experiences of nursing care.

This research revealed several factors that nurses find inhibiting when it comes to improving patient experiences of the quality of nursing care. Current nursing policy is heavily focussed on cost-effectiveness and transparency for external accountability, which creates a high administrative workload and pressure to increase productivity. However, despite all the registrations that take place for external accountability, the participating nurses stated that they do not monitor care results to improve their own practice. They felt they insufficient autonomy to influence this. They believe it is important to reflect upon and discuss nursing issues related to the quality of patient care, including patient experiences.

### Recommendation

Further research is recommended to examine whether the elements of a healthy work environment are statistically related to patient experiences in the Dutch healthcare setting. In the Netherlands, patient experiences are measured with the Consumer Quality Index (CQI) [6].

Nurses’ perceptions of their work environment are measured using the Essentials of Magnetism Tool II (EOMII) questionnaire [44]. Further research should focus on the statistical relations between CQI and EOMII.

## Abbreviations

ANCC: American Nurses Credentialing Center; PES-NWI: Practice environment scale of the nursing work index; EOMII: Essential of magnetism tool II; CQI: Consumer quality index; CAHPS: Consumer assessment of healthcare providers and systems.

## Competing interests

The authors declare that they have no competing interests.

## Authors’ contributions

RK participated in the design of the study, conducted the focus groups and analyses, and drafted the manuscript. BdB participated in the data collection (two focus groups) and revised the manuscript. DD participated in formulating the research questions, designing the study, and collecting and analysing the data (two focus groups), and helped to draft the manuscript. ALF participated in the design of the study and helped to draft the manuscript. All authors read and approved the final manuscript.

## Pre-publication history

The pre-publication history for this paper can be accessed here:

http://www.biomedcentral.com/1472-6963/14/249/prepub
